# Structural Determinants of Phenotypic Diversity and Replication Rate of Human Prions

**DOI:** 10.1371/journal.ppat.1004832

**Published:** 2015-04-14

**Authors:** Jiri G. Safar, Xiangzhu Xiao, Mohammad E. Kabir, Shugui Chen, Chae Kim, Tracy Haldiman, Yvonne Cohen, Wei Chen, Mark L. Cohen, Witold K. Surewicz

**Affiliations:** 1 Department of Pathology, Case Western Reserve University, Cleveland, Ohio, United States of America; 2 Department of Neurology, Case Western Reserve University, Cleveland, Ohio, United States of America; 3 National Prion Disease Pathology Surveillance Center, Case Western Reserve University, Cleveland, Ohio, United States of America; 4 Department of Physiology and Biophysics, Case Western Reserve University, Cleveland, Ohio, United States of America; Dartmouth Medical School, UNITED STATES

## Abstract

The infectious pathogen responsible for prion diseases is the misfolded, aggregated form of the prion protein, PrP^Sc^. In contrast to recent progress in studies of laboratory rodent-adapted prions, current understanding of the molecular basis of human prion diseases and, especially, their vast phenotypic diversity is very limited. Here, we have purified proteinase resistant PrP^Sc^ aggregates from two major phenotypes of sporadic Creutzfeldt-Jakob disease (sCJD), determined their conformational stability and replication tempo *in vitro*, as well as characterized structural organization using recently emerged approaches based on hydrogen/deuterium (H/D) exchange coupled with mass spectrometry. Our data clearly demonstrate that these phenotypically distant prions differ in a major way with regard to their structural organization, both at the level of the polypeptide backbone (as indicated by backbone amide H/D exchange data) as well as the quaternary packing arrangements (as indicated by H/D exchange kinetics for histidine side chains). Furthermore, these data indicate that, in contrast to previous observations on yeast and some murine prion strains, the replication rate of sCJD prions is primarily determined not by conformational stability but by specific structural features that control the growth rate of prion protein aggregates.

## Author Summary

Sporadic Creutzfeldt-Jakob disease (sCJD) represents ~90% of all human prion diseases worldwide. This neurodegenerative disease, which is transmissible and invariably fatal, is characterized by variable progression rates and remarkable diversity of clinical and pathological traits. The infectious sCJD prions propagating the pathology mainly in the brain are assemblies of abnormally folded isoform (PrP^Sc^) of a host-encoded prion protein (PrP^C^). The structure and replication mechanisms of human prions are unknown, and whether the PrP^Sc^ subtypes identified by proteolytic fragmentation represent distinct strains of sCJD prions has been debated. Here, we isolated sCJD prions from patients with two very distant phenotypes of the disease, compared their structural organization using recently developed biophysical techniques, and investigated their replication *in vitro*. Our data indicate that these sCJD prions are characterized by different secondary structure organization and quaternary packing arrangements, and that these structural differences are responsible for distinct prion replication rates and unique phenotypic characteristics of the disease. Furthermore, our analysis reveals that, contrary to previous observations for yeast prions, the replication tempo of sCJD prions is determined not so much by their conformational stability but by specific structural features that control the growth speed of prion particles.

## Introduction

**Fig 1:**
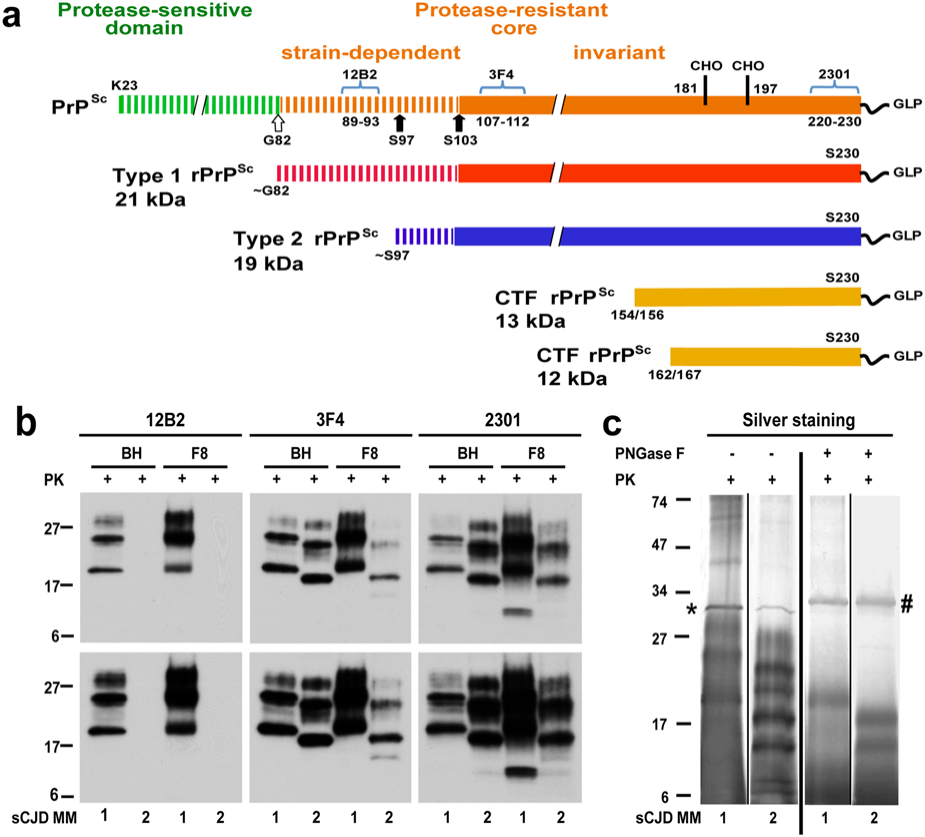
Schematic representation of PK-resistant fragments in rPrP^Sc^ corresponding to Type 1 (MM1) and Type 2 (MM2) sCJD prions and molecular characteristics of purified human rPrPSc used in structural studies. (a) Outline of classification of Type 1 and Type 2 human prions based on proteolytic fragmentation of PrP^Sc^ [5,52]. Major cleavage sites by PK are indicated by arrows; GLP—glycolipid; CHO- complex N-glycosylation chains. The codes above light blue brackets represent monoclonal antibodies used in differentiation of various domains of human prions, and the numbers below these brackets indicate linear epitopes recognized by these antibodies. (b) Distinct glycosylation patterns and electrophoretic mobilities of purified human Type 1 (MM1) and Type 2 (MM2) sCJD rPrP^Sc^ (homozygous for methionine (M) in codon 129) used in structural studies. To differentiate Type 1, Type 2 prions, and their C-terminal fragments, Western blots of purified rPrP^Sc^ (fraction 8; F8) from the brain homogenate (BH) of type MM1 and MM2 sCJD were developed with mAb 12B2 (epitope residues 89–93) [53], mAb 3F4 (epitope residues 107–112) [54], and rabbit polyclonal antibody 2301 (epitope residues 220–231) [55]. The lower panels correspond to prolonged exposure of the same WB to detect less abundant low molecular weight fragments of rPrP^Sc^. (c) Distinct fragmentation patterns of purified MM1 and MM2 sCJD prions in silver stained SDS-PAGE before and after deglycosylation with PNGase F. The symbols (*) and (#) indicate bands corresponding to PK and PNGase F, respectively. The molecular weights of marker proteins are in kDa.

Prions are a novel class of infectious agents that are composed solely of self-replicating misfolded protein aggregates [1]. In mammals, prions cause a group of invariably fatal and rapidly progressive neurodegenerative diseases, originally described as transmissible spongiform encephalopathies (TSEs) [1,2]. The most common of the human prion diseases is sporadic Creutzfeldt-Jakob disease (sCJD) [3], accounting for ~90% of all CJD cases worldwide [4]. One of the most intriguing features of these diseases is their vast phenotypic heterogeneity [1,4]. In patients homozygous for methionine in the *PRNP* gene, there are two major subtypes of sCJD: MM1 and MM2. These types differ with regard to the progression rate of the disease, pattern of proteinase K (PK)-resistant fragments of infectious prion protein aggregates PrP^Sc^, (Fig 1a), neuropathological characteristics of brain lesions, and transmissibility properties in transgenic mice [4–10].

A substantial progress has been made in recent years in prion research using laboratory rodent-adapted, cloned prion strains. These studies revealed, among others, that phenotypic variability of these model prions is directly linked to (and likely encoded in) structural differences of PrP^Sc^, and suggested that prion replication rates are inversely proportional to conformational stability of rodent PrP^Sc^ (as defined by the concentration of denaturant needed to dissociate/unfold PrP^Sc^ aggregates) [11,12]. By contrast, our understanding of the molecular basis of human prions such as those causing sCJD is far less advanced. These prions are present in human brain at a very low concentration (approximately 100-fold lower compared to that in a prion-infected rodent brain) and, thus, are much more difficult to purify and characterize. In fact, no direct structural data are available for PrP^Sc^ present in sCJD brains beyond the evidence that the N-terminus is variably resistant to denaturation and proteolytic digestion (Fig 1a) [5,7,13–17]. Even though earlier studies suggest that phenotypic diversity in human prion disease is somehow related to distinct PrP^Sc^ isoforms, conformational spectrum of these isoforms and the issue of strains of human prions are poorly understood, hindering efforts to develop generally accepted international classification of human prion disease. Moreover, the classical approach—isolation and definition of a full repertoire of sCJD prion strains in transgenic mice models with uniform genetic background—had not been successful due to the constrains imposed by the extensive phenotypic and genetic diversity of sCJD [4] and very long incubation time and/or limited transmissibility to transgenic mice [6,8–10]. The characterization of human prions is further complicated by the frequent co-existence of diverse prion particles [18,19] and prion adaptation and evolution in a new host [9,19].

To bridge some of these gaps, here we purified sCJD prions from two cases of phenotypically very distant sCJD types, determined their replication tempo as well as characterized structural organization using recently emerged approaches based on mass spectrometry-detected hydrogen/deuterium exchange. Our data provide direct experimental evidence that different phenotypes of sCJD are associated with structurally distinct PrP^Sc^ aggregates. Furthermore, these data suggest that, in contrast to the observations for murine prion strains [11,12], the replication rate of sCJD prions is not a pure function of conformational stability but is rather dictated by specific structural features of PrP^Sc^.

## Results and Discussion

From the collection of samples obtained from 340 patients with an unequivocal diagnosis of Type 1 (MM1) and Type 2 (MM2) sCJD, we selected one case that is representative of each neuropathology group (S1 Fig) and displayed ≥99% pure Type 1 or Type 2 proteinase K-resistant PrP^Sc^ (rPrP^Sc^), as detected by both conformation dependent immunoassay (CDI) and Western blots [13,14,20]. The disease duration in these representative cases, as well as biochemical characteristics of brain PrP^Sc^ associated with them (levels of total PrP^Sc^ and rPrP^Sc^, size of PrP^Sc^ particles, conformational stability of PrP^Sc^) correspond to the respective median values reported previously for each group [13,14] (Table 1).

**Table 1:** Source, biophysical characteristics, and replication rate of Type 1 and Type 2 sCJD prions.

Prion		Type	1		2	
**Parameter**		Unit		Mean ± S.E.M.		Mean ± S.E.M.
***PRNP* Gene**		codon 129	MM		MM	
**Age**		years	68		77	
**Sex**		F/M	F		F	
**Disease Duration**		month	5.4		11.2	
**PrP** ^**Sc**^		ng/ml		547 ± 62		1885 ± 159
**rPrP** ^**Sc**^		ng/ml		234 ± 10		911 ± 47
**[Gdn HCl]**_**1/2**_		M		3.0 ± 0.1		2.3 ± 0.1
**Mass**		Da		9–11 x 10^6^		≥14 x 10^6^
**Replication Rate**	**sPMCA**	n-fold		248 ± 13		133 ± 7
	**QuIC**	n-fold		915 ± 56		241 ± 15

The native prion particles containing rPrP^Sc^ from these two cases were purified for structural studies with a scaled up protocol we developed previously for purification of infectious and structurally intact Sc237 prions from Syrian hamster brains [21]. The Western blot patterns of purified MM1 and MM2 rPrP^Sc^ in the final fraction 8 (F8) and in the original brain homogenates (BH) were superimposable, documenting complete qualitative recovery of rPrP^Sc^ from brain homogenates (Fig 1b). As expected [22], the mass of unglycosylated fragments was ~21 kDa in Type 1 and ~19 kDa in Type 2 rPrP^Sc^, and Type 2 rPrP^Sc^ was not detectable with mAb 12B2 due to the missing N-terminal epitope (Fig 1b and S2b Fig). The 12–13 kDa C-terminal fragments were more abundant in Type 1 rPrP^Sc^ and detectable in Type 2 after longer exposure (Fig 1b). The silver-stained gels demonstrated the pattern of rPrP^Sc^ corresponding to the major bands on Western blots, and the isolated rPrP^Sc^ was ~90% pure (Fig 1c). These patterns were highly reproducible upon purification of rPrP^Sc^ from different cortical areas of the same brain (S2 Fig).

**Fig 2:**
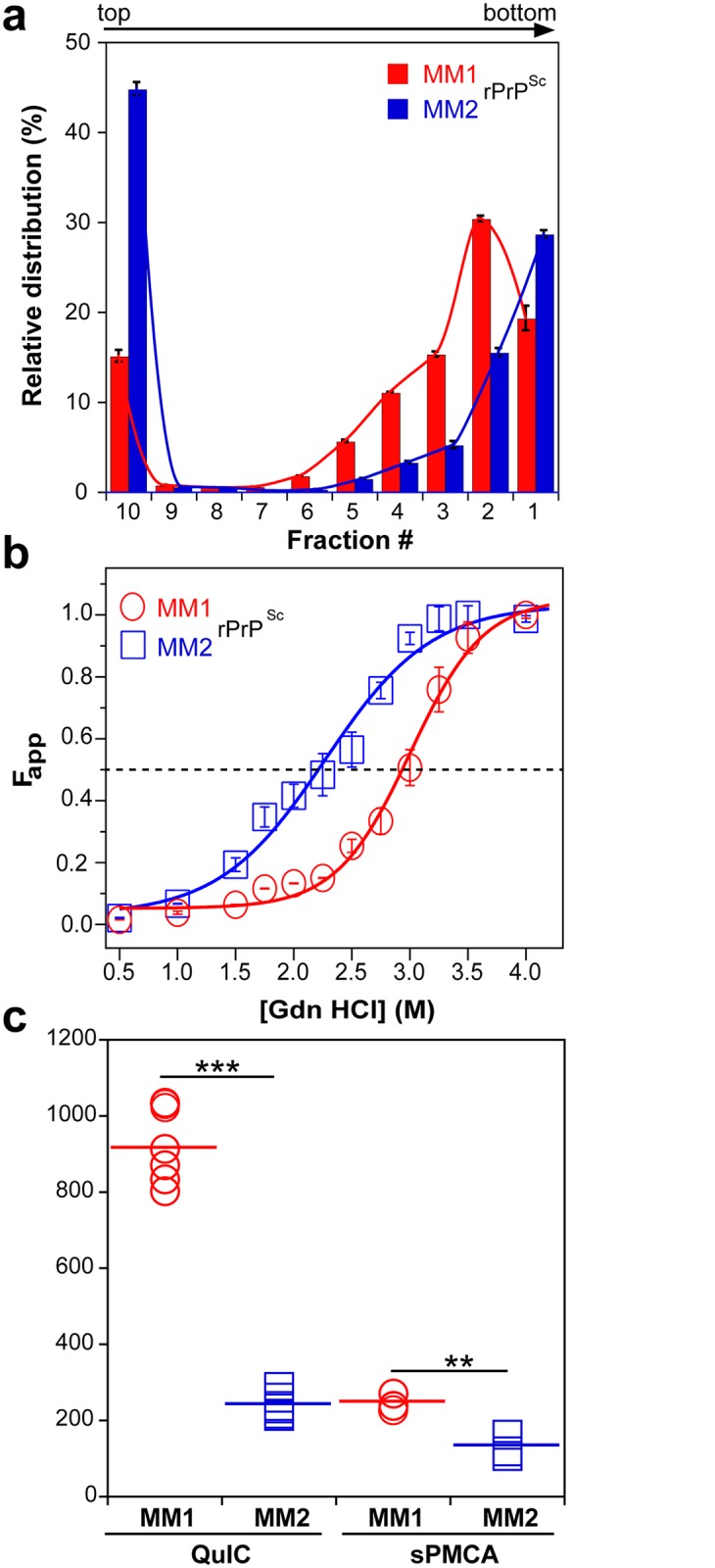
Sedimentation velocity, conformational stability, and seeding potency of isolated sCJD prions. (a) Distinct sedimentation velocity profiles of MM1 and MM2 prions. The samples were fractionated by ultracentrifugation in sucrose gradient and fractions were collected from the bottom of the tubes and analyzed for rPrP^Sc^ by CDI. The bars represent average ± SEM; CDI was performed on each sCJD sample in triplicate. (b) The conformational stability of MM1 and MM2 rPrP^Sc^. The curves represent best fit to a sigmoidal function. The values of apparent fractional change (Fapp) are mean ± SEM obtained from four batches of purified MM1 and MM2 prions, each determined in triplicate measurements. (c) Amplification of MM1 and MM2 sCJD prions by QuIC using recombinant human PrP(23–231, 129M) substrate and by sPMCA using brain homogenate of Tg mice expressing human PrP^C^ (129M). The amplification index is the ratio between the concentration of PrP^Sc^ before and after PMCA measured with CDI. The data points represent results of six QuIC and three sPMCA experiments, each measured in triplicate with CDI. The mean values are indicated by horizontal lines. *** P<0.001, ** P<0.005 determined by ANOVA.

To investigate the prion size, we separated sCJD prion particles according to sedimentation velocity in sucrose gradient [13]. Consistent with previous data, the peak sedimentation velocity of MM1 rPrP^Sc^ was found to be substantially slower than that of MM2 rPrP^Sc^ [13] (Fig 2a). Based on calibration with standard proteins [13], we estimate that the majority of MM1 rPrP^Sc^ particles have a molecular mass of 9-11x10^6^ Da (~380–460 monomers), whereas the respective value for MM2 rPrP^Sc^ particles is ≥14x10^6^ Da (≥600 monomers) (Fig 2a and Table 1).

Using CDI, we compared the conformational stability of rPrP^Sc^ obtained from different brain cortex areas in four independent purification rounds from each sCJD case (Fig 2b). The average stability of MM1 rPrP^Sc^ against denaturation by GdnHCl was significantly higher than that of MM2 rPrP^Sc^, with GdnHCl concentration corresponding to midpoint denaturation of 3.0 and 2.3 M, respectively (Fig 2b and Table 1).

Next, we assessed the seeding efficacy (amplification index) of MM1 rPrP^Sc^ and MM2 rPrP^Sc^ *in vitro* using two different methods, QuIC and sPMCA. The amplification index (potency) of different seeds is expressed as a ratio between the concentration of the PrP^Sc^ conformers produced with PMCA or QuIC divided by the concentration of PrP^Sc^ in the seed after subtracting the background obtained in control unseeded samples. Detailed protocols of these methods and control experiments showing lack of spontaneous prion protein conversion in the unseeded reactions have been described previously [13]. In both assays, the seeding efficacy of MM1 rPrP^Sc^ was markedly higher compared to that of MM2 rPrP^Sc^ (Fig 2c and Table 1). This higher replication efficacy of MM1 prions from the case selected for the present structural studies is consistent with ~4-fold higher median replication potency of MM1 prions compared to MM2 prions we previously observed (using both QuIC and sPMCA techniques) for prions from ten MM1 and ten MM2 sCJD cases (Supplemental S2 Fig in [13]). These data in vitro are also in accord with available bioassay data that demonstrate higher transmission rates and significantly shorter incubation times of MM1 sCJD prions in transgenic mice expressing human PrP^C^ (129M) or human/mouse PrP^C^ chimeras [8,9]. The higher replication efficiency of the conformationally more stable MM1 rPrP^Sc^ is both intriguing and unexpected, as some previous experiments with mouse prion strains suggest that there is an inverse correlation between prion replication rate and conformational stability of total PrP^Sc^ (i.e., the less stable conformers should replicate faster) [11,12]. Our present data indicate that this previously suggested relationship does not apply to sCJD rPrP^Sc^.

Clearly, understanding the molecular basis of phenotypic variability in sCJD requires structural characterization of PrP^Sc^ that goes beyond relatively crude assays such as proteolytic fragmentation followed by Western blotting or conformational stability measurements. This is a challenging task because most of the methods developed for structural studies of protein aggregates are not applicable to brain-derived PrP^Sc^, as they require isotopic labeling or introduction of other spectroscopic probes. However, new opportunities in this regard are offered by two mass spectrometry based methods: backbone amide hydrogen/deuterium exchange coupled with mass spectrometry (HXMS) [23] and histidine hydrogen/deuterium exchange mass spectrometry (His-HXMS) [24]. Here, we used these two methods for structural comparison of MM1 rPrP^Sc^ and MM2 rPrP^Sc^.

The HXMS method measures the rate of H/D exchange of protein backbone amide hydrogen atoms. Since the exchange rates are much faster for protein segments that are unstructured as compared to those that are involved in H-bonded structures such as α-helices or β-sheets, these measurements provide a sensitive tool for conformational analysis. This approach, which we recently successfully used for structural analysis of strain-specific differences in murine prions [23], is especially useful for studying amyloids and related protein aggregates, as the exchange rates within the β-sheet cores of these aggregates are exceptionally slow [25–30].

**Fig 3:**
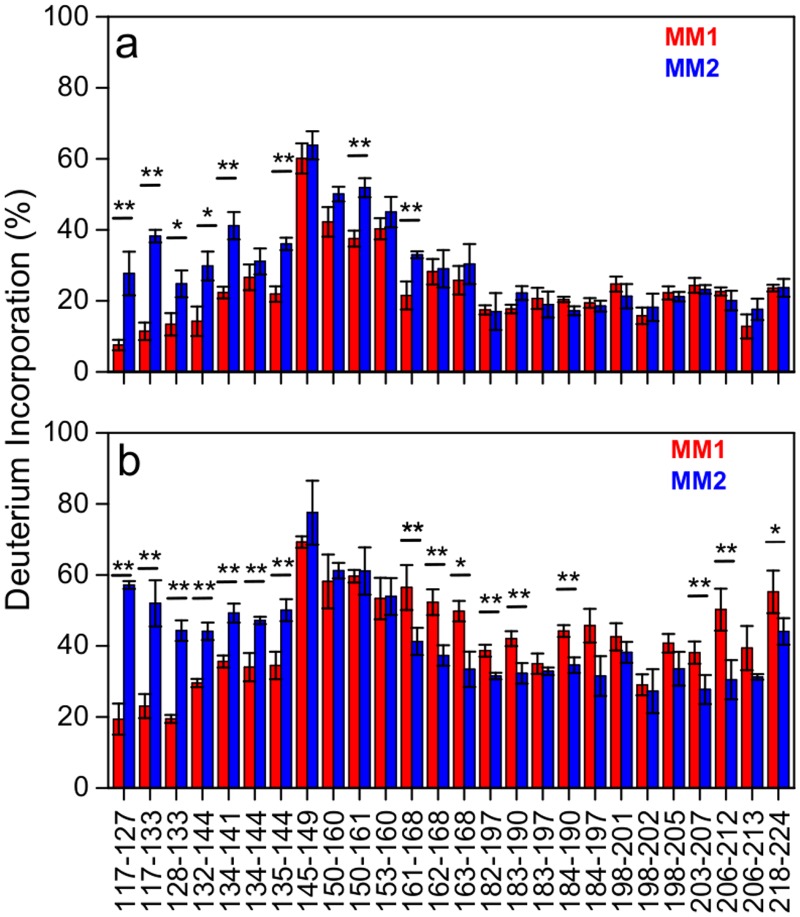
Deuterium incorporation for peptic fragments derived from MM1 rPrP^Sc^ (red) and MM2 rPrP^Sc^(blue). (a) 5 min incubation in D_2_O. (b) 240 h incubation in D_2_O. Error bars indicate standard deviation (3 independent experiments). *, P<0.05; **, P<0.02.

The first step in HXMS analysis is the generation of peptic fragments that can be separated by ultrahigh performance liquid chromatography (UHPLC) and identified by MS. Both for MM1 and MM2 rPrP^Sc^, we were able to identify 27 peptic fragments that give rise to MS spectra with a signal-to-noise ratio sufficient for reliable calculation of deuterium incorporation. These fragments (some of them partially overlapping) cover ~85% of the C-terminal region 117–224, with the only significant gap for the segment 169–181 that contains one of the glycosylation sites (likely due to a very low concentration of peptic fragment(s) derived from the nonglycosylated component of rPrP^Sc^). No peptic fragments could be analyzed from the N-terminal region up to residue 116, presumably due to the ragged N-terminus of human rPrP^Sc^. The extent of deuterium incorporation for MM1 rPrP^Sc^ and MM2 rPrP^Sc^ after 5 min and 240 h incubation in D_2_O is shown in Fig 3. For both rPrP^Sc^ types, the region of relatively little protection against deuterium incorporation maps to residues ~145–160. This is in striking contrast to murine prion strains studied to date, in which case this central region is characterized by high degree of protection against H/D exchange [23]. Thus, it appears that the ~145–160 region of sCJD prions is structurally less ordered than the same region in cloned murine prion strains.

In contrast to similar protection against deuterium incorporation in the ~145–160 regions of MM1 and MM2 rPrP^Sc^, there are substantial differences in other parts of rPrP^Sc^ corresponding to distinct sCJD phenotypes. This is especially evident for the 117–144 region, as peptic fragments derived from this part of MM1 rPrP^Sc^ consistently show higher degree of H/D exchange as compared to those corresponding to the same region in MM2 rPrP^Sc^. The difference is particularly striking for the 117–133 region, in which case the degree of deuterium incorporation after 240 h exchange is 2.5–3 fold lower for MM1 rPrP^Sc^, indicating markedly higher level of structural order in this part of MM1 rPrP^Sc^ as compared to MM2 rPrP^Sc^. An opposite trend is observed for the C-terminal region ~161–224, where higher protection against H/D exchange is observed for MM2 rPrP^Sc^ (Fig 3). Altogether, these data clearly demonstrate substantial structural differences between rPrP^Sc^ corresponding to two different phenotypes of sCJD. The resolution of HXMS alone is not sufficient to propose any specific structural model that could account for these differences. However, within the context of the frequently considered model based on the parallel in-register β-structure motif [31,32], region-specific differences in resistance to H/D exchange observed between MM1 PrP^Sc^ and MM2 PrP^Sc^ could likely reflect factors such as different proportions in these regions of residues involved in β-strands and loops between them and/or packing differences between individual β-strands. As in the case of murine prions [23], high level of protection against H/D exchange in the C-terminal region of sCJD PrP^Sc^ is not compatible with the structural model proposing that residues ~89–175 form left-handed β-helices, with the C-terminal region retaining the native-like α-helical conformation of PrP^C^ [33]. However, the present data alone do not exclude the possibility that the entire PK-resistant region of PrP^Sc^ could form a β-helix-like structure.

Structural properties of sCJD prions were further probed using the recently developed approach of His-HXMS which measures the rate of H/D exchange of C2 protons in histidine side chains [34–36]. Information provided by this method is complementary to that obtained from amide HXMS measurements: while amide HXMS probes protein structural organization and dynamics at the level of the polypeptide backbone, His-HXMS probes the microenvironment (water accessibility) of specific His side chains [34–36]. As shown in a recent study with recombinant prion protein amyloid fibrils, the latter approach can be particularly useful in probing quaternary structure of ordered protein aggregates, providing information about the packing arrangement and interfaces between β-sheets [31].

**Fig 4:**
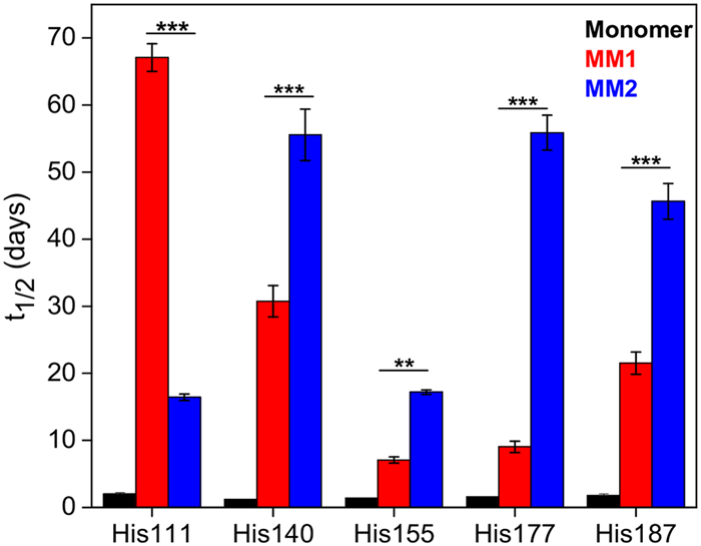
Histidine H/D exchange for monomeric PrP^C^ (black), MM1 rPrP^Sc^ (red) and MM2 rPrP^Sc^ (blue). The parameter t_1/2_ represents the half-time of exchange reaction for individual His residues. Error bars indicate standard deviation (3 independent experiments). **, P<0.01; ***, P<0.001.

There are six His residues in the PK-resistant region of human PrP^Sc^ (His99, His111, His140, His150, His177 and His187). MS signal for the peptide fragment containing His99 was too weak to allow reliable measurements. However, high quality H/D exchange data could be obtained for five other His residues. In the native structure of the PrP^C^ monomer, all these His side chains are fully exposed to water. Thus, as expected for unprotected histidines [34,35], the half-times of exchange are about 2–3 days. In the rPrP^Sc^ structures, these half-times are substantially longer, indicating that all His side chains are located in at least partially water-protected environment (Fig 4). However, the degree of this protection for individual His side chains varies greatly between MM1 rPrP^Sc^ and MM2 rPrP^Sc^. For example, in MM1 rPrP^Sc^, His177 is still in a relatively water accessible environment (exchange half-time of 9 days), whereas in MM2 PrP^Sc^, this side chain is much more protected from water (exchange half-time of 56 days). An opposite situation is observed for His111, in which the environment of the side chain is much more water-protected in the structure of MM1 rPrP^Sc^ than that of the MM2 counterpart (exchange half-times of 67 and 16 days, respectively).

Recent crystallographic studies with amyloidogenic peptides identified two types of interfaces between β-sheets in amyloid structures: one that is highly water protected with strong interdigitation of side chains (“dry” steric zipper) and one that is more accessible to water [37,38]. Within this context, the differences in water exposure of individual His side chains observed between MM1 rPrP^Sc^ and MM2 rPrP^Sc^ could be explained by distinct packing arrangements of β-sheets in these two structures (i.e., the same His side chain being in dry or wet interface depending on the rPrP^Sc^ type). It should be noted, however, that even for the most protected His side chains in rPrP^Sc^, the exchange half-times are substantially shorter than those recently observed in synthetic amyloid fibrils prepared from the recombinant PrP (hundreds of hours), suggesting that steric zippers in brain-derived rPrP^Sc^ might be less perfect than those in synthetic amyloid fibrils. This is not entirely surprising given that rPrP^Sc^ particles contain glycosylated isoforms, and glycans may interfere with packing between β-sheets.

Experiments with two strains of yeast prion [PSI^+^] demonstrated that, in this case, the critical determinant of the strength of prion phenotype is the susceptibility of prion aggregates to fragmentation (that creates new ends for monomer recruitment), with the less stable structure corresponding to the stronger phenotype [39]. This fragmentation and maintenance of the yeast prion state *in vivo* is believed to be mediated by the molecular chaperone Hsp104 [39,40]. Even though there are no known mammalian homologs of the disaggregating chaperone HsP104, the general hypothesis that less stable prions are more virulent has been adopted in the field of mammalian prions, and this model appeared to be supported by studies *in vivo* with some rodent prion strains [11,12,41], even though the results of these studies could also be explained by strain-dependent differences in prion clearance rates. Furthermore, an inverse correlation was found between the replication tempo *in vitro* and the conformational stability of the protease-sensitive sCJD PrP^Sc^ (but not the protease-resistant component, rPrP^Sc^) [13]. However, data for some other rodent prion strains appear to be inconsistent with this model [42,43]. The picture is further complicated by the fact that there are no reliable direct assays to probe fragmentation susceptibility of PrP^Sc^ aggregates, and their stability is typically assessed by measuring resistance to denaturation with SDS or chaotropes; the relationship between the latter property and fragility is not necessarily straightforward.

Our present data clearly demonstrate that, in contrast to the observations for some murine prions, lower conformational stability of sCJD rPrP^Sc^ does not result in higher replication rate of these prions. Thus, at least in the case of human prions, conformational stability of rPrP^Sc^ (as defined by resistance to denaturation with SDS or chaotropes) is definitely not a reliable predictor of the incubation period of the disease. Importantly, our structural studies allowed us to identify substantial differences between the molecular organization of MM1 and MM2 rPrP^Sc^, both at the level of the polypeptide backbone as well as the quaternary packing arrangements. As shown in our recent study with recombinant PrP amyloid fibrils[24], the differences in packing between β-sheets may result in distinct conformational stabilities. However, it appears that it is not the conformational stability *per se* that controls the replication rate of rPrP^Sc^, as the observed faster replication of MM1 sCJD prions when compared to MM2 counterparts would imply a paradoxical scenario in which higher stability of rPrP^Sc^ results in a faster replication tempo. Instead, our data strongly suggest that distinct replication rates of MM1 and MM2 sCJD prions are dictated by specific structural features of corresponding rPrP^Sc^ aggregates, features that control the intrinsic growth rate of these aggregates (i.e., the rate of templated conformational conversion of the PrP^C^ substrate). Thus, the balance of factors controlling strain-specific replication tempo of sCJD prions appears to be diametrically different from that described for yeast prions [PSI^+^] that are associated with aggregation of Sup35 protein. In the latter case, the intrinsic elongation rate of Sup35 amyloid fibrils Sc4 corresponding to the stronger (faster replicating) prion phenotype is slower than that of fibrils Sc37 corresponding to the weaker phenotype, but this is more than compensated by lower stability (and thus higher effective concentration of ends) for Sc4 fibrils [39]. By contrast, the faster replicating strain of sCJD prion is characterized by higher conformational stability, implying that, in this case, the dominant factor in controlling the replication tempo is not prion stability but the intrinsic growth rate (i.e., the rate of the conversion of PrP^C^ monomers).

Considerable structural differences between type 1 and type 2 PrP^Sc^ in sCJD are especially intriguing given frequent coexistence of these two prion strains in affected individuals [20]. Whether this strain coexistence is the result of a primordial spontaneous misfolding or conformational evolution due to the template flipping during passage through cells expressing different post-translationally modified PrP^C^ remains to be determined [19]. It should also be noted that type 1 and type 2 sCJD prions represent only a small fraction of the spectrum of human prions. It is likely that the structural variability among PrP^Sc^ corresponding to different familial forms of human prion diseases might be even larger than the extent of structural differences described herein for type 1 and type 2 sCJD prions.

## Materials and Methods

### Ethics statement and clinicopathologic characteristics of sCJD cases

All procedures were performed under protocols approved by the Institutional Review Board at Case Western Reserve University. In all cases, written informed consent for research was obtained from patients or legal guardians and the material used had appropriate ethical approval for use in this project. All patients’ data and samples were coded and handled according to NIH guidelines to protect patients’ identities. We selected two representative subjects from a group of 340 patients with definitive diagnosis of sCJD. The criteria for inclusion were: (1) availability of clinical diagnosis of CJD according to WHO criteria [44,45] and clearly determined and dated initial symptoms upon neurologic examination to ascertain the disease duration; (2) methionine homozygous at codon 129 of the human prion protein (PrP) gene (PRNP); (3) unequivocal classification as pure Type 1 or Type 2 sCJD according to WB pattern; (4) unequivocal classification of pathology as definite Type 1 or 2 at the National Prion Disease Pathology Surveillance Center (NPDPSC) in Cleveland, Ohio; (5) demographic data distribution within 95% confidence interval of the whole group, resulting in no difference between selected cases and the whole group in any of the statistically followed parameters.

### Purification of prions from sCJD human brains

The purification of rPrP^Sc^ from human brains was performed as described previously for 263K prions from Syrian hamster brains [21] with the following additional steps. The partially purified samples containing ~10 μg of human PrP^Sc^ were resuspended in PBS, pH7.4 containing 2 mM CaCl_2_ and 2% Sarkosyl, sonicated in a sonication bath (3 x 5 s), and incubated with 70 μg/ml of Collagenase (Worthington Biochemical Corporation) with shaking at 600 rpm in Eppendorf Thermomixer for 4 h at 37°C. After adding MgCl_2_ to a final concentration of 5 mM, the samples were incubated with 50 IU/ml of Benzonase (Novagen/EMP) for additional 1 h at 37°C, followed by 1 h incubation with 100 μg/ml of proteinase K (Amresco, Solon, OH/ Invitrogen) at 37°C. The PK was blocked with protease inhibitor (PI) cocktail containing 0.5mM PMSF, and 5 μg/ml of aprotinin and leupeptin, respectively. The pellet obtained after centrifugation (30 min, 18,000 x g, 4°C) in Allegra centrifuge equipped with F2402H rotor was resuspended in 400 μl of 10% NaCl containing 1% Sarkosyl and PI cocktail, and spun again. The final pellet was resuspended in PBS containing 2% Sarkosyl and PI cocktail (1:1000, v/v), and delipidated overnight with four volumes of Methanol/Chloroform (2:1, v/v) at -20°C. Finally, the sample was collected by centrifugation, resuspended in water containing 0.1% Sarkosyl and stored at -80°C.

### Physicochemical properties and molecular characteristics of purified sCJD prions

The purified rPrP^Sc^ was analyzed by SDS PAGE followed by silver staining and/or western blots, and by conformation-dependent immunoassay (CDI). The latter assay was performed as described previously [6,14] with the following minor modifications. First, we used white Lumitrac 600 High Binding Plates (E&K Scientific, Santa Clara, California) coated with mAb 8H4 (epitope 175–185)[46] in 200 mM NaH_2_PO_4_ containing 0.03% (w/v) NaN_3_, pH 7.5. Second, aliquots of 20 μl from each fraction containing 0.007% (v/v) of Patent Blue V (Sigma) were directly loaded into wells of white strip plates prefilled with 200μl of Assay Buffer (Perkin Elmer, Waltham, Massachusetts). Finally, the captured PrP was detected by a europium-conjugated [47] anti-PrP mAb 3F4 (epitope 107–112) and the time-resolved fluorescence (TRF) signals were measured by the multi-mode microplate reader PHERAstar Plus (BMG LabTech, Durham, North Carolina). The recHuPrP(90–231,129M) and PrP(23–231,129M) used as a calibrant in the CDI was prepared and purified as described previously [48]. The conformational stability of rPrP^Sc^ was determined with CDI as described previously [14,47] and the raw CDI signal was converted into the apparent fractional change and fitted by least square method with a sigmoidal transition model to determine GdnHCl concentration where 50% of PrP^Sc^ is unfolded ([Gdn HCl]_1/2_) [14]. The sedimentation velocity and mass of sCJD prions was determined with calibrated sucrose gradient ultracentrifugation as described [13].

### Replication rate of sCJD prions measured in vitro

The Quaking-induced Conversion (QuIC) [49] and sonication-driven serial Protein Misfolding Cyclic Amplification (sPMCA) [50] procedures were performed essentially as described previously [13,14]. Briefly, rhuPrP(23–231,129M) used as a substrate in QuIC was expressed, purified, and refolded to α-helical conformation as described previously [48], and its initial concentration was calculated from absorbance at 280 nm using the molar extinction coefficient 56650 M^-1^cm^-1^. The stock of rhuPrP(23–231) in 10 mM sodium acetate buffer, pH 4.0, was pretreated with 12 mM HCl [rhuPrP:HCl ratio (v/v) of 1:3.9] for 7.5 min and immediately diluted to a final concentration of 0.1 mg/ml into the reaction buffer composed of 20 mM NaH_2_PO_4_, 130 mM NaCl, pH 6.9, and containing 0.1% SDS, 0.1% Triton X-100, and 1:5000 (v/v) N2 (Invitrogen, Carlsbad, California). The QuIC was performed with final volume of 100 μl per well in a sterile V-bottom, low-binding polypropylene 96-well plate (VWR, Arlington Heights, Illinois) equipped with a 3 mm diameter PTFE bead (Fisher Scientific, Pittsburgh, Pennsylvania) in each well. The aliquots of sCJD brain homogenates were diluted into the complete QuIC reaction buffer to obtain final 10^-4^ dilution of sCJD prions, and the plates were sealed with sterile AxyMat Silicone Sealing Mat (VWR, Arlington Heights, Illinois). The QuIC was performed in samples seeded with sCJD PrP^Sc^ at 55°C for 20 hrs in an Eppendorf Thermomixer (Eppendorf, Hauppauge, New York) set for 1 min shaking at 1400 rpm, followed by 1 min incubation. The reaction was stopped by adding to each well 50 μl of PBS (pH 6.9) containing 3% (w/v) Sarkosyl and Proteinase K (PK; Amresco, Solon, Ohio) to obtain the final Sarkosyl concentration of 1% (w/v) and PrP/PK ratio of 10:1 (w/w). The plates were incubated for 1 h at 37°C with shaking at 1200 rpm on the Eppendorf Thermomixer with 1 min intervals. The PK was blocked in each well with protease inhibitors (0.5 mM PMSF, 5 ug/ml of aprotinin and leupeptin) and the PK-resistant PrP was measured with CDI [13,14].

Sonication-driven serial Protein Misfolding Cyclic Amplification (sPMCA) of sCJD samples was performed as described [50] with the following modifications. Human PrP^Sc^ was replicated using brains of transgenic mice overexpressing human PrP with methionine at position 129 [51]. The 10% brain homogenates from sCJD patients were diluted 1000-fold into 10% normal brain homogenate and 100 μl was transferred into 0.2 ml PCR tubes equipped with 2.38 mm diameter PTFE ball (K-mac Plastics, Wyoming, Michigan). Tubes were positioned on an adaptor placed on the plate holder of a microsonicator (Misonix Model 3000, Farmingdale, New York) programmed to perform cycles of 60 min incubation at 32°C followed by a 30 s pulse of sonication set at 80% power. Samples were incubated, without shaking, and immersed in the water of the sonicator bath. After a round of 24 cycles, a 10 μl aliquot of the amplified material was diluted into 90 μl of normal transgenic mouse brain homogenate and a new round of 24 PMCA cycles was performed. This procedure was repeated four times to reach a final 10^6^-fold dilution of the initial sCJD brain homogenate, and the replication rate was calculated from PrP^Sc^ content measured before and after sPMCA with CDI [13,14,19].

### Backbone amide hydrogen/deuterium exchange mass spectrometry experiments (HXMS)

To initiate deuterium labeling, 10 μl aliquots of purified sCJD rPrP^Sc^ (~1.8 μg) were collected by centrifugation (21000×g, 30 min, 4°C) and added to 100 μl of 10 mM phosphate buffer (pH 7.3) in D_2_O. After incubation at room temperature for different time periods, samples were collected by centrifugation and dissociated into monomers by adding 20 μl of ice cold 100 mM phosphate buffer (pH 2.5) containing 7 M GdnHCl and 0.1 M Tris (2-carboxyethyl) phosphine hydrochloride. After 5 minutes incubation (~0°C), the solution was diluted 10 times with ice cold 0.05% trifluoracetic acid and digested for 5 min with pepsin as described previously [23]. The peptic fragments were collected in a C18 trap column (Symmetry C18 NanoEase, Waters, USA), washed to remove salts, and eluted on a UPLC BEH-C18 HPLC column (Waters, USA) using a gradient of 2–45% acetonitrile at a flow rate of 23 μl/min. Peptides separated on the column were analyzed by an LTQ Orbitrap XL mass spectrometer (ThermoElectron, San Jose, CA). To minimize back-exchange, both the trap and the analytical column were placed in a cooled chamber (~2°C) integrated with a LEAP TriValve system (LEAP Technologies, USA). The extent of deuterium incorporation in each peptic fragment was determined from mass spectra (with a correction for back-exchange) as described previously [23].

### Histidine hydrogen/deuterium exchange (His-HXMS) experiments

For these measurements, samples of purified sCJD rPrP^Sc^ from human brain (~3 μg) were suspended in D_2_O buffer (10 mM sodium phosphate, 10 μM EDTA, 50 μM Pefabloc, 1 ug/ml Aprotinin, pH 9.0). After incubation for 5 days at 37°C, samples were collected by centrifugation and deglycosylated with PNGase F. To obtain fragments containing single His residues, samples were then digested with immobilized pepsin, followed by digestion with immobilized trypsin. Finally, the peptic fragments were separated on an UPLC column and analyzed by mass spectrometry as described above for HXMS experiments. The pseudo-first-order rate constant (k) of His hydrogen exchange reaction was determined by the equation: k = -ln{1-[**(**(R(t)—R(0))/**(**(1 + R(t)—R(0))] x 1/P}/t, where P is the fractional D_2_O content in the solvent, R is the ratio of M+1/M isotopic peak of a given peptide before (time = 0) and after the H/X reaction (time = t). The half-life (*t*_1/2_, days) of His exchange reaction was calculated using the equation: *t*_1/2_ (day) = ln2/k/24, where k (hour^-1^) is the rate constant at the alkaline conditions (pH = 9) [34,35].

## Supporting Information

S1 Fig Distinct neuropathologic characteristics of the occipital neocortex in Type 1 (a, c) and Type 2 (b, d) sCJD cases homozygous for methionine in codon 129 of PRNP gene and used as a source of human prions in structural studies. (a, b) Spongiform degeneration. Typical fine vacuole-type spongiform changes with diffuse small round vacuoles in Type 1 (a) contrast with large coarse fused vacuoles in Type 2 sCJD (b). (c, d) PrP^Sc^ deposition. Dispersed punctate (synaptic-type) PrP^Sc^ deposition in occipital cortex of Type 1 sCJD (c) contrasts with large plaque-like deposits frequently associated with vacuols in Type 2 sCJD (d). Scale bar is 50 μm.
(PDF)

S2 Fig Highly reproducible electrophoretic patterns of MM1 and MM2 sCJD prions purified from different cortex areas of the same human brain. (a) The silver staining after SDS-PAGE of ~300 ng of purified rPrP^Sc^ from different cortical areas of the same sCJD Type 1 (lanes I-III in the left panel) and Type 2 (lanes I-IV in the right panel) case before and after deglycosylation. Asterisk (*) and double dagger (#) point to the bands of PK and PNGase F, respectively. (b) Western blot analysis of the purified human MM1 and MM2 sCJD prions before and after deglycosylation. The lower panels are from the same WB taken after longer exposure to detect less abundant low mass fragments of rPrP^Sc^. The molecular weights of the marker proteins are in kD.
(PDF)
