# Supplementary material for: Structural Determinants of Phenotypic Diversity and Replication Rate of Human Prions
Source: PLoS Pathog. 2015 Apr 14;11(4):e1004832. doi: 10.1371/journal.ppat.1004832 (PMC4397081; doi:10.1371/journal.ppat.1004832)
Supplement: S1 Fig — (a, b) Spongiform degeneration. Typical fine vacuole-type spongiform changes with diffuse small round vacuoles in Type 1 (a) contrast with large coarse fused vacuoles in Type 2 sCJD (b). (c, d) PrPSc deposition. Dispersed punctate (synaptic-type) PrPSc deposition in occipital cortex of Type 1 sCJD (c) contrasts with large plaque-like deposits frequently associated with vacuols in Type 2 sCJD (d). Scale bar is 50 μm. (PDF) [file ppat.1004832.s001.pdf]

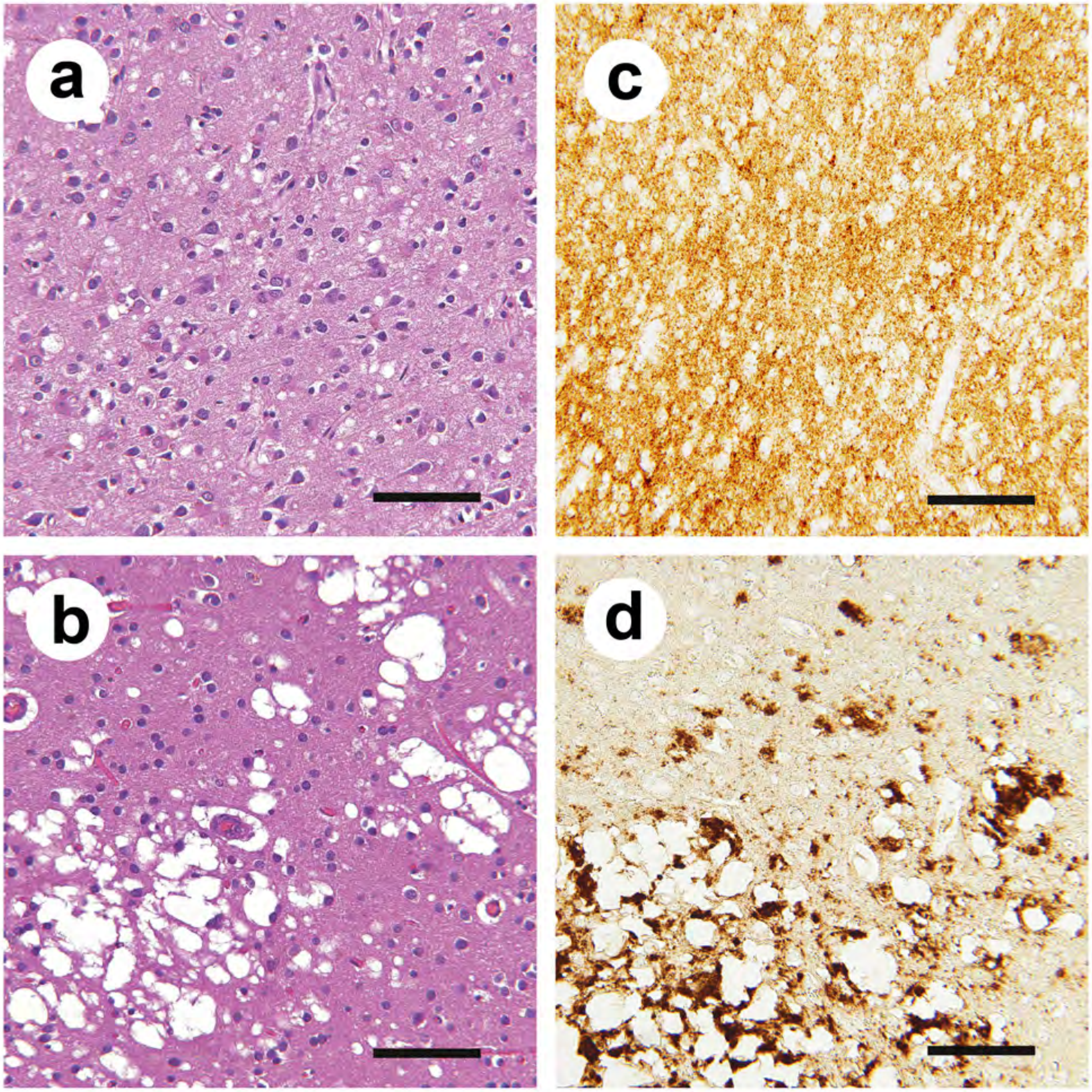

Figure S1. Distinct neuropathologic characteristics of the occipital neocortex in Type 1 (**a, c**) and Type 2 (**b, d**) sCJD cases homozygous for methionine in codon 129 of PRNP gene and used as a source of human prions in structural studies. (**a, b**) Spongiform degeneration. Typical fine vacuole-type spongiform changes with diffuse small round vacuoles in Type 1 (**a**) contrast with large coarse fused vacuoles in Type 2 sCJD (**b**). (**c, d**) PrP^{Sc} deposition. Dispersed punctate (synaptic-type) PrP^{Sc} deposition in occipital cortex of Type 1 sCJD (**c**) contrasts with large plaque-like deposits frequently associated with vacuols in Type 2 sCJD (**d**). Scale bar is 50 µm.
