# Supplementary material for: Structural Determinants of Phenotypic Diversity and Replication Rate of Human Prions
Source: PLoS Pathog. 2015 Apr 14;11(4):e1004832. doi: 10.1371/journal.ppat.1004832 (PMC4397081; doi:10.1371/journal.ppat.1004832)
Supplement: S2 Fig — (a) The silver staining after SDS-PAGE of ~300 ng of purified rPrPSc from different cortical areas of the same sCJD Type 1 (lanes I-III in the left panel) and Type 2 (lanes I-IV in the right panel) case before and after deglycosylation. Asterisk (*) and double dagger (#) point to the bands of PK and PNGase F, respectively. (b) Western blot analysis of the purified human MM1 and MM2 sCJD prions before and after deglycosylation. The lower panels are from the same WB taken after longer exposure to detect less abundant low mass fragments of rPrPSc. The molecular weights of the marker proteins are in kD. (PDF) [file ppat.1004832.s002.pdf]

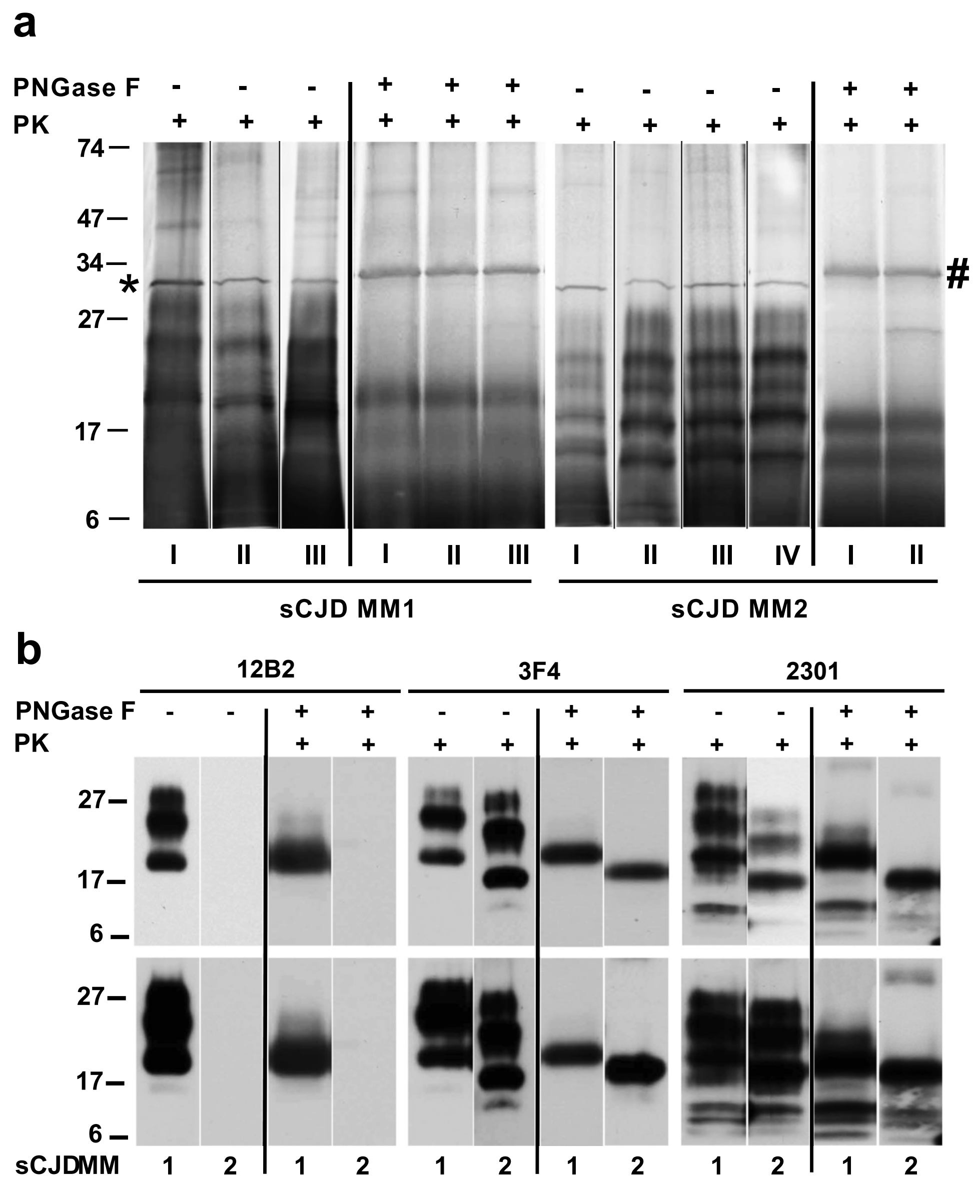

Figure S2. Highly reproducible electrophoretic patterns of MM1 and MM2 sCJD prions purified from different cortex areas of the same human brain. (**a**) The silver staining after SDS-PAGE of ~300 ng of purified $rPrP^{Sc}$ from different cortical areas of the same sCJD Type 1 (lanes I-III in the left panel) and Type 2 (lanes I-IV in the right panel) case before and after deglycosylation. Asterisk (*) and double dagger (#) point to the bands of PK and PNGase F, respectively. (**b**) Western blot analysis of the purified human MM1 and MM2 sCJD prions before and after deglycosylation. The lower panels are from the same WB taken after longer exposure to detect less abundant low mass fragments of $rPrP^{Sc}$. The molecular weights of the marker proteins are in kDa.
